# Colocalization methods in pituitary tumorigenesis aged-related in MEN1 KO and wild type mice

**Published:** 2014

**Authors:** C Stancu, M Coculescu

**Affiliations:** * Department of Endocrinology, “Carol Davila” University of Medicine and Pharmacy, Bucharest, Romania

**Keywords:** immunohistochemistry, electron microscopy, confocal microscopy, pituitary, MEN1

## Abstract

**Background**. Colocalization analysis of confocal fluorescence and electron microscopy (EM) are important tools to detect the expression of multiple anterior pituitary hormones within the same cell. Heterozygous (Men1+/-) mice developed pituitary tumors, mostly reported somatolactotrophinomas and ACTH secreting pituitary adenomas but also nonfunctioning tumors.

**The aim** of the study was to run immunohistochemistry protocols to study colocalization of pituitary hormones in newborn mice in tumoral and non-tumoral tissue in MEN1-KO and wild type control mice.

**Methods**: Pituitary samples from nine Men1+/- mice, 29-34 days old male mice (n=8) and one year old (n=1) and control group, four new born (1,5 days old) wild type (*mus musculus*) mice were analyzed by immunofluorescence immunohistochemistry (GH, PRL, gonadotrophs) to find hormonal colocalization in pituitary cell. Moreover, pituitaries were embedded in LRGold for immunogold labeling technique (GH, PRL, gonadotrophs and alpha-SU) also.

**Results**: Pituitary tumors, immunoreactive only for PRL were found in three MEN1 – KO mice. No sign of pituitary hyperplasia was found in MEN1-KO. MEN1-KO non-tumoral pituitary displayed similar immunoreactivity to wild type pituitary. Colocalization studies revealed individual cells PRL-FSH immunoreactive and GH-FSH immunoreactive in the non-tumoral tissue from MEN1-KO mice and in wild type pituitaries respectively but no colocalization in the tumoral tissue.

**In conclusion**, colocalization is a feature of neonate mouse pituitary but not in adults. The MEN1-KO pituitary tumors were prolactinomas and unlike non-tumoral pituitary tissue of MEN1-KO, displayed no PRL-FSH colocalization.

## Introduction

Pituitary pathology started at beginning of 20th century with the routine hematoxylin and eosin (HE) staining but soon, the need for a more detailed characterization appeared. As soon as anterior pituitary hormones were characterized, development of specific antibodies were used not only for biological assays but also for detailed pathology. In the late 70’s, immunohistochemical details of pituitary gland realized an important step forward in the pathology diagnosis. Moreover, the concept one cell one hormone theory, initially very solid, were shown to be inaccurate. In the same time, technological progress made the ultrastructural characterization available. Various pituitary cells were characterized according to secretory granules (dimension, abundance, distribution). These details were further used in the pituitary pathology for the characterization of pituitary adenomas. In the last 25 years, the development of immunohistochemical techniques was tremendous. Specific antibodies for hormone subunit, receptors, enzymes or proliferation markers came from basic research into clinical practice at a very high speed. Nowadays, routine immunohistochemistry includes for pituitary, all anterior pituitary hormones as well as Ki67 immunostaining as a proliferation index.

Fluorescence microscopy added some advantages in pituitary pathology assessing a greater detail of good sensitivity and possible evaluation of in vivo cells in cell cultures by using calcium sensitive fluorescent dyes (FURA2) [**1**].

However, immunohistochemistry for single hormones showed just a static image of a complex movie, which involved the transdifferentiation from one cell type to another, proliferation of stem cells into various cell lines even in adult pituitary. In order to make a step forward into this complex mechanism of pituitary cell regulation, colocalization studies are important: they allow the obtaining of clear evidence that in a single cell we are able to find several different hormones, transcription factors or receptors. In this way, colocalization IHC is an important tool in order to detect pituitary cell complex processes. 

Immunocytochemistry (IHC) by using ultrastructural resolution can be performed by using antibodies bound to gold particles. Immunogold techniques can characterize secretory vesicles in terms of hormonal content. In electron microscopy (EM) IHC one of the biggest problems is related to the balance/compromise between immunoreactivity and structure: the better the fixation, the lower the antigenicity. Expression of multiple anterior pituitary hormones within the same cell is a characteristic of the fetal pituitary [**2**] but can also appear in pituitary adenomas. An acromegalic patient with a GH-secreting macroadenoma in whom the immunogold technique revealed a colocalization of GH and FSH in some tumor cells, with an ultrastructure similar to normal gonadotroph cells was reported [**3**]. 

A study was designed to investigate whether PRL/GH and FSH/LH/alpha subunit may be colocalized in mammotrophs and somatotrophs or possibly other types of cells used MEN1 mouse model generated through homologous recombination of the mouse homolog MEN1. The MEN1 gene encodes menin, which is a tumor suppressor. Heterozygous (Men1+/-) mice were generated by targeted deletion of exons 1 and 2, a 1.9 kb 50 sequence that encompassed the untranslated region, promoter and alternative exons 1, and a w1 kb sequence of the 50 region of intron 2 of the Men1 gene. These sequences were replaced by a neomycin resistance gene in the embryonic stem cells. Heterozygous mice develop features remarkably similar to those of the human disorder. Men1+/- mice aged >12 months developed tumors of anterior pituitary, consistent with an age-related penetrance for the condition. More than 75% of the pituitary tumors contained prolactin, GH and ACTH, and the remainder were considered nonfunctioning tumors [**4**].

The *Principles of Laboratory Animal Care* (NIH publication no. 85–23) was followed and the study was in accordance with the UK Animals (Scientific Procedures) Act 1986. 

Wild type mice, aged 1.5 days old male mice were terminally anesthetized by ip injection of 3 mg sodium pentobarbital (Sagatal, Rhone Merieux, France) and perfused through the heart with heparinized saline (0.9% NaCl and 10 U/ml heparin); pituitary glands were collected after cervical dislocation in 4% paraformaldehyde in PBS (sodium phosphate buffer). Hematoxylin and eosin (HE) procedure was performed for the correct identification of anterior pituitary tissue. After that, all pituitaries were cut in half; one half was fixed in a mixture of 4% paraformaldehyde and 0.5% glutaraldehyde (primary fixative) and prepared for electron microscopy by standard methods. Briefly, segments were post-fixed in osmium tetroxide (1% w/v in 0.1 m sodium phosphate buffer) contrasted with uranyl acetate (2% w/v in distilled water), dehydrated through increasing concentrations of methanol (70–90 %) and embedded into acrylic resin - LRGold (Agar Scientific (UK), Stansted, UK) under UV polymerization at 20°C. The other halves were washed in 0.1m acetate buffer twice, block strain in 2% uranyl acetate in acetate buffer for 1 h and cryoprotected in 30% sucrose until sank with 4% paraformaldehyde overnight.

Pituitary samples from MEN1 KO were fixed in a mixture of 3% paraformaldehyde, 0.05% glutaraldehyde in 0.1molar phosphate buffer, except for pituitary from MEN1 KO mice aged one year old fixed in a mixture of 3% paraformaldehyde, 3% glutaraldehyde in 0.1molar phosphate buffer.

**Immunofluorescence**

Frozen pituitaries were embedded in Tissue Tek, then cut at 12 micrometers, the sections were thaw – mounted on gelatincoated glass slides, then dried at RT (room temeprature).

The first steps were to establish an optimal immunofluorescence procedure, choosing the primary and secondary antibodies and finding the appropriate dilutions. The antibodies must have no cross-reactivity. In order to choose the best signal/ noise ratio serial dilution of primary antibodies were tested: rabbit anti rat GH: 1:200, 1:400, 1:800 (original is 1:1.67), rabbit anti human PRL:1:200,1:400, 1: 800 (original is 1:5); rabbit anit mouse PRL 1:800, 1:1600 (original is 1:50), rabbit anti rat alpha subunit 1:400, 1:800 (original is 1:6). Rabbit polyclonal or mouse monoclonal antibody to FSH/LH were used. Primary antibodies were located with either fluorescein anti rabbit IgG or Texas red anti mouse IgG linked secondary antibodies. We tested also whether increasing the dilution of the fluorescein – or texas red coupled secondary antibody improving the specificity of staining (i.e. we might expect only about 20% of the cells to stain strongly for FSH or LH; there is always the problem that most cells will stain for GH). 

On frozen sections, we did immunofluorescence protocols: simple and double staining (colocalization).

Sections were rinsed twice with PBS, non-specific antibody binding was blocked by preincubation with 5% fetal calf serum in PBS, rinsed twice with PBS, incubated for 4 h with the primary antibody, washed in PBS three times for 5 min then incubated for 1 h with a fluorescein coupled (FITC) secondary antibody, washed PBS, 2 x 5 min and mounted in Vectashield fluorescence mounting medium (Vector Laboratories, Burlingame, CA, USA). We tested whether by using a double step antibody procedure (biotin, avidin) we could improve the specificity. For this, we did the primary antibody stage as mentioned above, then washed with PBS (no protein) then incubated with biotinylated anti mouse or anti-rabbit as appropriate for the primary antibody, then washed again with PBS, incubated with fluorophore-labelled avidin, washed again, and mounted.

Images of double-stained sections are subjected to background correction.

The protocol of Bendayan was used for double labeling experiments; GH- FSH/LH and PRL –FSH/LH, we sequentially applied the protocol for each antigen by using GH rabbit anti rat, PRL rabbit anti mouse primary antibody, washed PBS 3x5 min, incubated with secondary antibody biotinylated anti rabbit for 1 h, washed with PBS 3x5 minute, fluorescein avidin 1:100 for 1 h, washed 3x5 minute, incubated with the second monoclonal antibody (rabbit anti rat FSH/LH), washed 3x5 minute and incubated with Texas red anti-mouse IgG 1:100 for 1 h. Sections were mounted in Vectashield mounting medium without propidium iodide. The sections were examined on a Leica laser confocal microscope and images acquired at 1024x1024 pixels resolution.

**Immunoelectron microscopy (EM)**

**Fig. 1:**
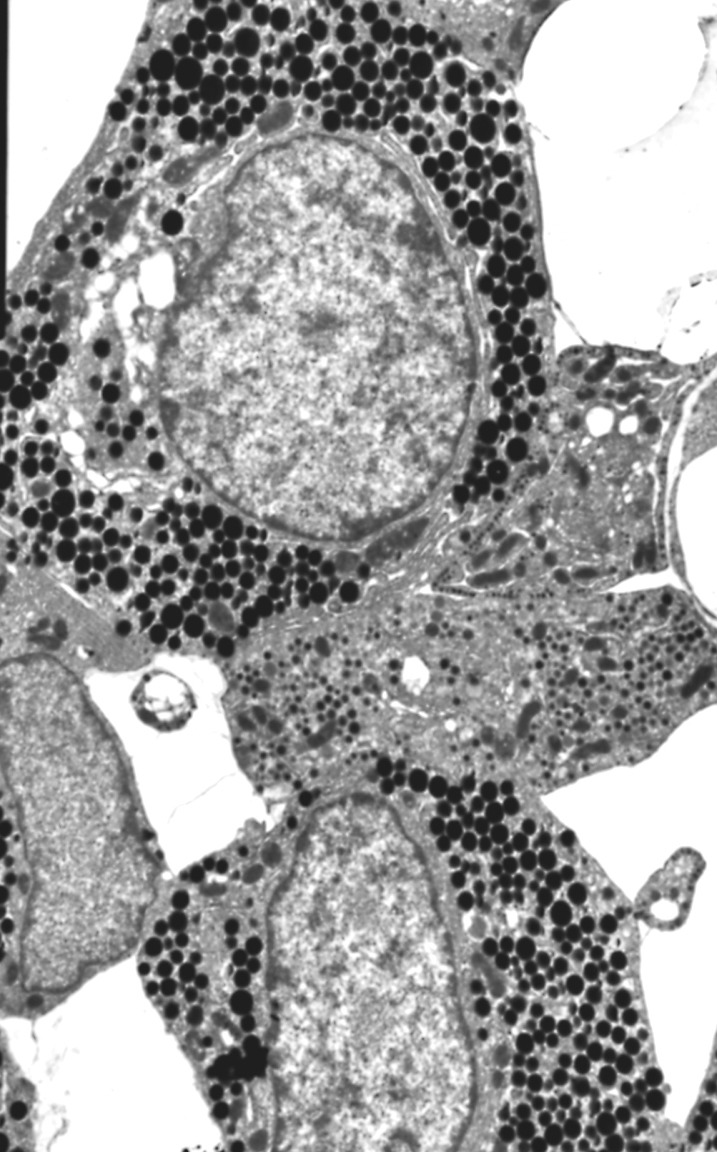
Wild type pituitary, EMSections were viewed with a JEM-1010 transmission electron microscope (JEOL USA Inc., Peabody, MA, USA). Relevant images were recorded on film and analyzed for type of secretory vesicles and gold particles.

Immunocytochemistry at ultrastructural were performed on semithin sections of specimens embedded in acrylic resins. The semithin sections were verified to assess the quality of the fixation and to select the area to be investigated by electron microscopy (**Fig. 1**). 

The LR-Gold blocks were cut in a pyramid form, stained with the toluidine blue solution (applied for 30 seconds) and examined under a light microscope to identify the region of the block containing the sample. Then, ultrathin sections (1 micrometer) were obtained, a diamond knife was used and sections were mounted on a nickel grid and immunogold labeled for GH, PRL, FSH, LH and alpha subunit. Briefly, grids were soaked in water, rinsed twice in PBS, non-specific binding was blocked by incubation in 4% BSA in PBS, rinsed twice in PBS, incubated for 2 h at 25oC with the primary antibody, rinsed twice in PBS, incubated with protein A gold (PAG) 15 nm or goat anti rabbit IgG 5 nm for 15 min, rinsed twice in PBS, contrasted with uranyl acetate and lead citrate, rinsed in PBS, then in distilled water and air dried. Of course, first we tested what concentration was needed to locate and we used: anti GH rabbit anti rat 1:400, anti PRL rabbit anti mouse 1:400, anti alfaSU rabbit anti rat 1:400, anti FSH anti human mouse monoclonal 1: 10, anti LH anti human mouse monoclonal 1:10.

Colocalization on electron microscopy were not posible because I did not available other size of PAG, different from PAG 15 nm, goat anti rabbit IgG 5 nm did not work, no particle was found even in background.

## Results

**Fig. 2:**
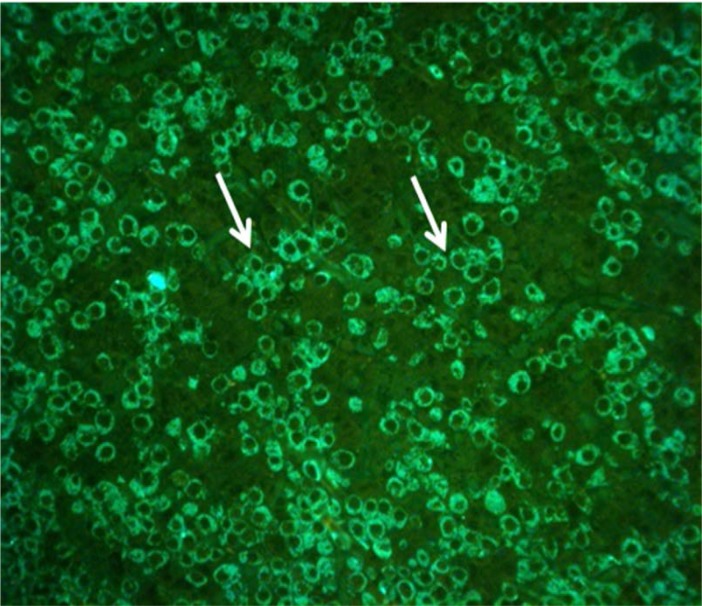
Wild type pituitary section, immunofluorescence (fluorescein) staining for GH, 1: 800

New born wild type pituitary sections were used to set up adequate immunostaining protocols for pituitary hormones for colocalization studies. Our protocols worked well for both immunofluorescence and electron microscopy staining. With appropriate dilution of primary antibodies, good cellular differentiation and good background were obtained (**Fig. 2**). 

 Endocrine cells in sections taken systematically from different depths of the embedded tissue were identified based on their secretory granule populations (shape, electron density, size and distribution). Immunogold labeling for PRL, FSH/LH/alpha subunit and GH was performed to assist with the identification of lactotrophs, gonadotrophs and somatotrophs, respectively.

**Fig. 3:**
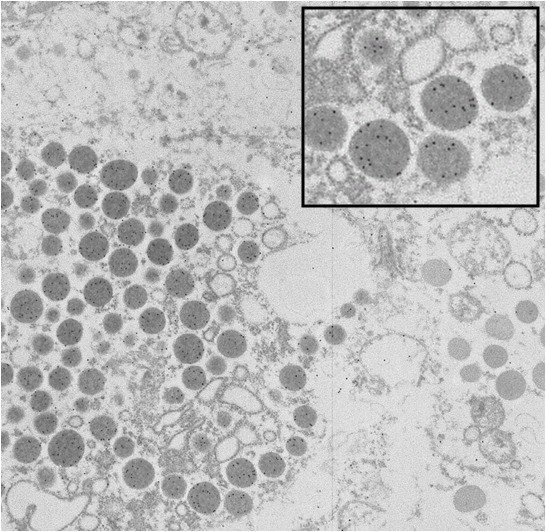
Wild type pituitary for GH 15 nm immunogold, EM

**Fig. 4:**
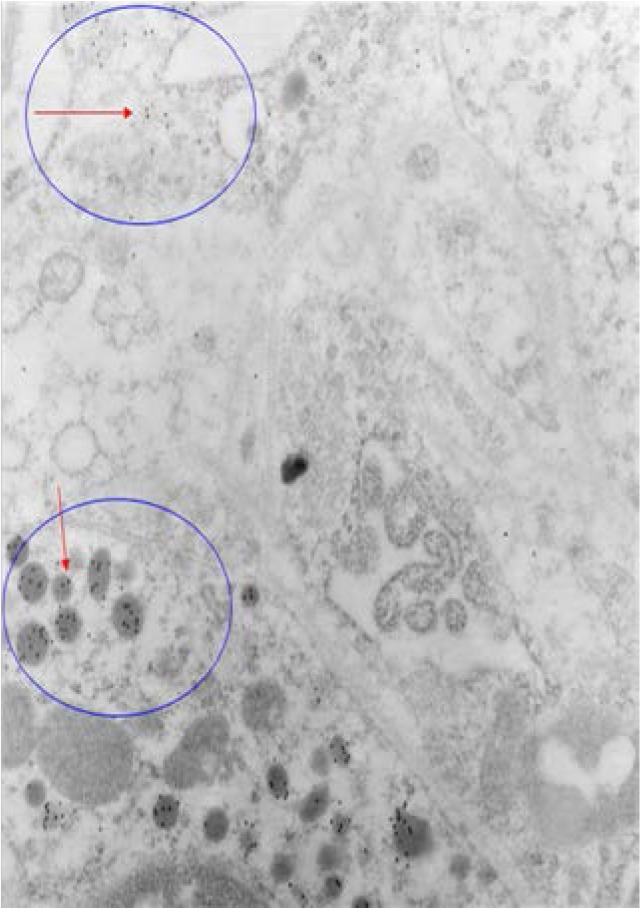
Wild type pituitary mouse showed PRL in two types of lactotrophs cell

In this wild type pituitaries, secretory granules in GH cells and PRL cells showed a positive immunoreaction to their respective antisera. GH cell population are well represented with spherical electrondense secretory granules (**Fig. 3**) and PRL cell population showed two types of lactotrophes, cell with electron-dense vesicles and cells containing electron-lucent vesicles (**Fig. 4**).

**Fig. 5:**
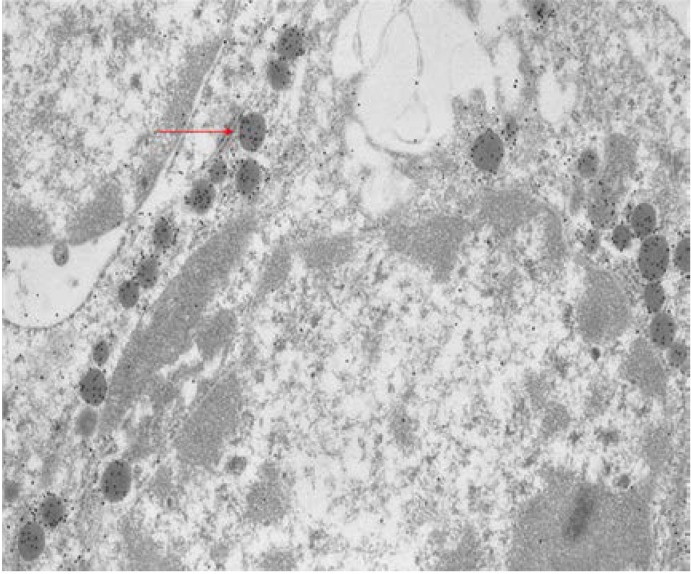
Wild type pituitary mouse – for alpha subunit 15 nm immunogold

**Fig. 6:**
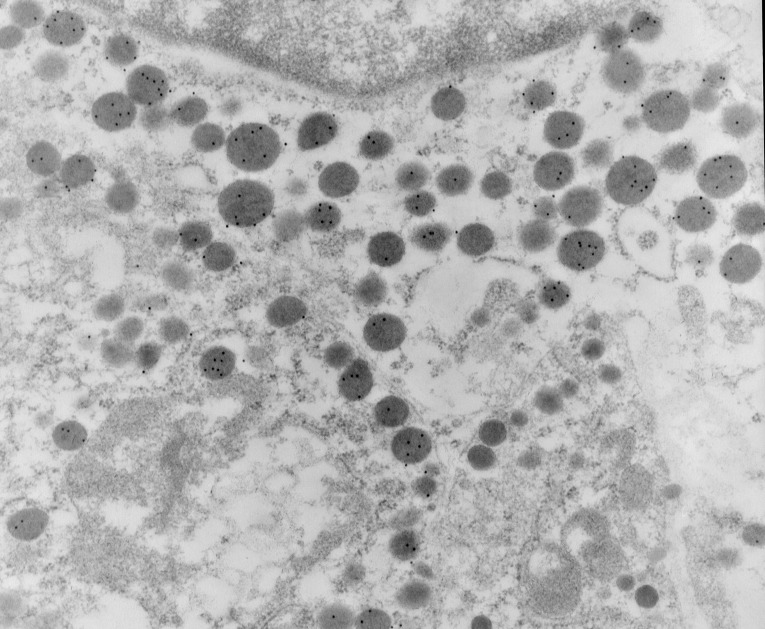
Wild type pituitary mouse for LH 15 nm immunogold

Gonadotrophic secretion in wild type mice evidenced the EM level by immunoreactivity for LH, FSH and for alpha subunits (**Fig. 5**, **6**).

Colocalization studies on fluorescence microscopy revealed individual cells GH-FSH immunoreactivity in newborn wild type pituitaries. Colocalization on electron microscopy was not possible because the other size of PAG, different from PAG 15 nm was not available. We have tried goat anti rabbit IgG 5 nm but it did not work, no particle was found, not even in the background.

**Fig. 7:**
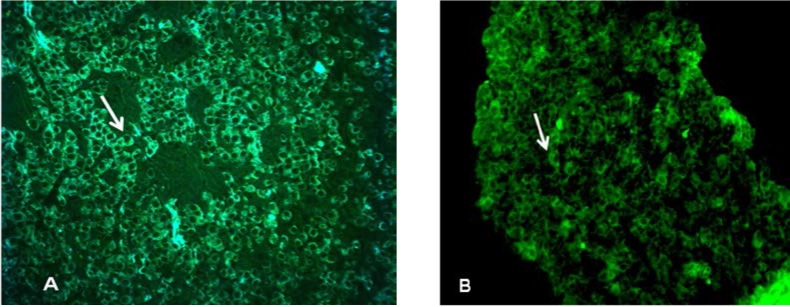
In the non – tumoral MEN1 mouse, the cells showed good immunoreactivity for GH (A) and for prolactin (B)

**Fig. 8:**
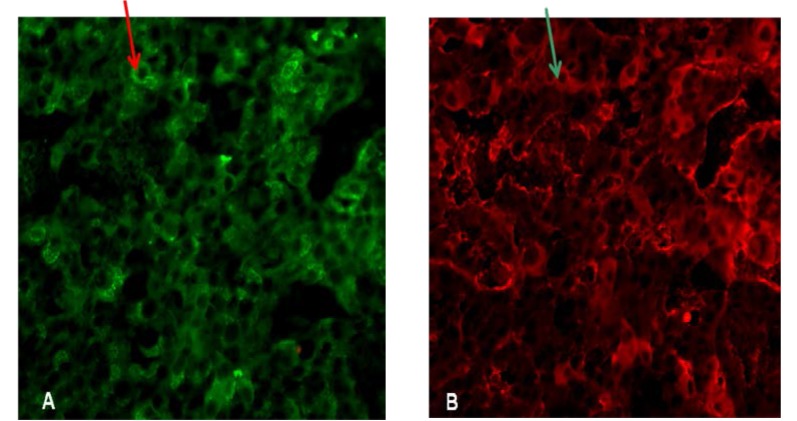
Non-tumoral MEN1 KO pituitary – double staining PRL (fluorescein) and FSH (texas red)

MEN1+/- non-tumoral pituitary mice displayed similar immunoreactivity to wild type pituitary. The immunofluorescence studies showed pituitary abundant immunofluorescence for GH and PRL (**Fig. 7**) and colocalization studies revealed PRL-FSH immunoreactivity (**Fig. 8**) in new born, MEN1-KO non-tumoral. 

Pituitary sections from MEN+/- showed at EM, a good immunostaining for GH, no vesicles with prolactin and a few immunogold alpha subunit particles (one or two per vesicles) without background. A few lactotrophe cells and many ACTH cells we had identified at ultrastructural level, but FSH immunoreactivity was weak, as was LH to a much lesser extent than GH and PRL, due to fewer immunoreactive cells and fewer secretory vesicles inside the cells. ACTH immunogold stain protocol was not done.

**Fig. 9:**
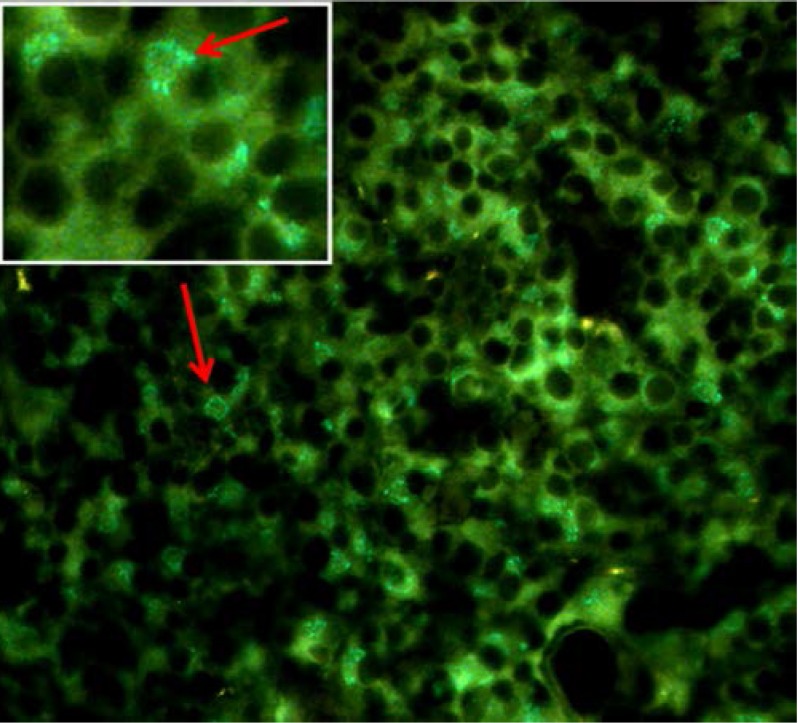
MEN1 KO tumor, stains positively for LH in 29-34 days old mice

There was no sign of pituitary hyperplasia in MEN1-KO. At EM in three MEN1-KO mice (two of them aged 29, 34 days old and one of them at one year old) mitochondria in clusters showed what explained the oncogenic changes. These pituitary tumors stains appeared positive for PRL but also for LH in 29-34 days old mouse (**Fig. 9**).

**Fig. 10:**
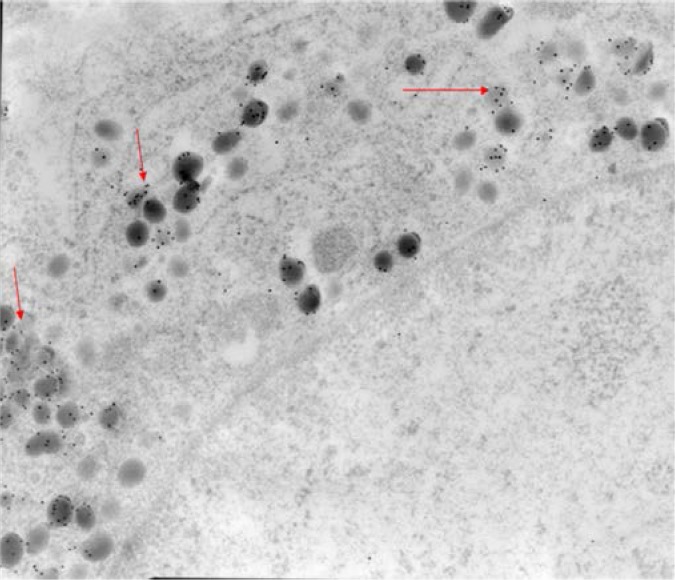
Tumoral MEN1 1 year old showed immunoreactivity for PRL

Pituitary tumors MEN1 mice, one year old were immunoreactive for PRL, but not for other pituitary hormones. Electron microscopy revealed several types of pituitary tumor cells: some with electron-dense vesicles were characteristic for somatotroph or mammosomatotroph cells, while cells containing electron-lucent vesicles appeared closer to gonadotroph ultrastructure (**Fig. 10**).

**Fig. 11:**
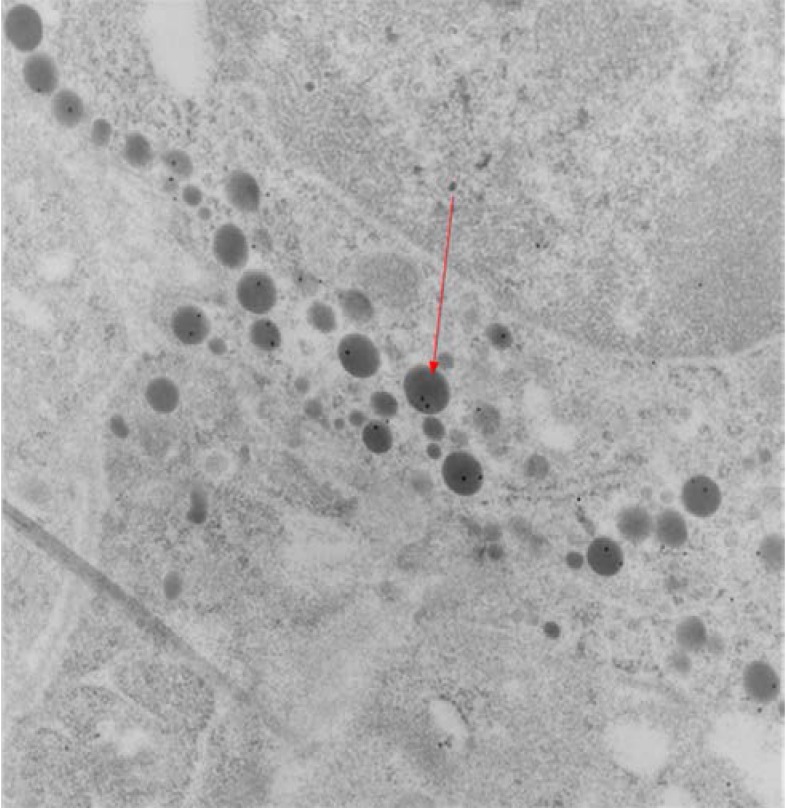
Tumoral MEN1- 1 year old showed weak immunoreactivity for alpha subunit

Tumoral MEN1- 1 year old showed weak immunoreactivity for alpha subunit (1-2 particles in vesicle) (**Fig. 11**). There is no background, this means it is not by chance. Because the tissues were fixed with a high concentration of glutaraldehyde, the antigenicity for alpha subunit could be low.

## Discussion

Successful colocalization experiments require good knowledge and experience in basic immunohistochemical techniques. When two or more antibodies labeled with different fluorophores are used to stain cells, multiple protein distributions can be visualized simultaneously. The fixation and optimal staining of cells are important for troubleshooting potential problems.

Fluorescence microscopy has long been used to characterize cellular organization and is based on the property of fluorescent molecule whereby they absorb the light of a particular wavelength and emit light at longer wavelengths.

To observe fluorescence in most biological specimens by microscopy, they first must be stained with a fluorochrome. The ultimate goal of staining specimens with fluorochromes is to get a specific fluorescence that makes certain areas of a specimen clearly visible. Antiserum quality plays a major role in the success of immunofluorescence investigations. Various animals have been used successfully for antibody production, including mammals and birds. The preferred mammal for antibody production is the rabbit because of its good immune response and convenient size and handling. Monoclonal antibodies are particularly attractive for many fluorescent antibody studies because they offer a very high degree of specificity. Good dilutions of antibodies, no cross-reactivity and steps of protocol are important for a relevant immunostaining [**5**].

A good differentiation of cells with high dilutions of monoclonal primary antibodies from rabbit was obtained.

Electron microscopy by using antibody conjugated with gold particles permits the high resolution detection and localization that depends on the antigen - recognition specificity of the primary antibodies, the preservation of the antigenicity of the antigens and the ability of the antibodies to penetrate the cell so that they are able to bind to the antigens [**6**]. In our experience with MEN1 tumor tissue that was fixed with 3% glutaraldehyde we obtained beautiful tissue morphology but low immunoreactivity.

For double localization, the principles are just the same as for immunofluorescence studies but instead of using different fluorophores different size of gold particles can be used and so, antigens can be detected simultaneously. Unfortunately, we could not demonstrate the colocalization found in fluorescent microscopy with EM colocalization.

The theory one cell, one hormone for cells of pituitary gland was modified because it was shown that some bihormonal cell contains 2 hormones. In Sprague-Dawley and Long Evans pituitaries, co-expression of PRL-GH, PRL-TSH, and TSH-GH [**7**] was found in both nontumorous and adenomatous pituitaries by electron microscopy and the immunogold double-labeling technique. Pituitary tumors that produce GH and PRL have long being recognized to derive from mammosomatotrophs; they are the most common type of mixed adenomas and they may co-express α sub-unit glycoprotein. In one study combining data from cultured pituitary tumor cells and immunohistochemistry, glycoproteins were often expressed in somatotroph tumors more frequently than in lactotroph, corticotroph and null cell adenomas. Also, intact TSH was secreted by more somatotroph cells cultures (15/23) as compared to other tumor types [**8**]. We found hormonal colocalization in newborn normal mouse pituitary wild type and heterozygous MEN1+/-. In pituitary tumor, we found only a suggestion that there are vesicles with alpha subunit, because the tissue was fixed in high glutaraldehyde concentration of that decrease antigeniticity. For the immunoelectron microscopy, fixation is one of the most important steps in sample preparation due to the need to preserve as much biochemical reactivity as possible, the mixture of formaldehyde and a low concentration of glutaraldehyde is the most generally accepted one for immunoelectron microscopy. A high concentration of glutaraldehyde is a fixative strong enough to have a good ultrastructural preservation [**9**] . 

The most important idea in this study is that colocalization is present in newborn normal mice and also in MEN+/- very young and, in tumoral tissue, at adult age there is no colocalization.

The relation between multihormonal progenitor cells and tumoral cells still remains to be established.

## Conclusions 

Our studies confirmed hormonal colocalization in pituitaries from newborn mice. GH-FSH colocalization is a feature of normal mouse pituitary. The MEN1-KO pituitary tumors have similar immunoreactivity with human prolactinomas. Unlike non-tumoral pituitary tissue of MEN1-KO, these tumors display no PRL-FSH colocalization. 

**Acknowledgements**

Prof. John Morris is gratefully acknowledged for the coordination of my research activity in Department of Physiology, Anatomy & Genetics, University of Oxford, UK.

This work was performed in the frame of PhD student program and ”Gr. Coculescu“ neuroendocrinology scholarship from the Romanian Psychoneuroendocine Society.
